# Case Report: Anlotinib Therapy in a Patient With Recurrent and Metastatic RAIR-DTC Harboring Coexistent TERT Promoter and BRAF^V600E^ Mutations

**DOI:** 10.3389/fonc.2021.626076

**Published:** 2021-03-24

**Authors:** Yanjun Su, Shaohao Cheng, Jun Qian, Min Zhang, Tuanli Li, Ying Zhang, Chang Diao, Ling Zhang, Ruochuan Cheng

**Affiliations:** Department of Thyroid Surgery, The First Affiliated Hospital of Kunming Medical University, Kunming, China

**Keywords:** differentiated thyroid carcinoma, anlotinib, therapy, BRAFV600E, TERT

## Abstract

We describe a case of recurrent and metastatic radioactive iodine-refractory differentiated thyroid cancer (RAIR-DTC) treated with anlotinib in this report. The patient was randomized to placebo initially, after disease progressed at C8 (C is the treatment cycle), the patient was referred to the open label therapy of anlotinib, 12 mg p.o. daily with a 2-week on/1-week off regimen. Partial response was achieved at C2 with anlotinib treatment. To date, over 37 months of progression-free survival (PFS) has been achieved. Adverse effects were tolerable and manageable in this patient. Molecular characterization revealed coexistent C228T mutation of TERT promoter and BRAF^V600E^ mutations. Favorable clinical outcome in this patient suggests that anlotinib might provide a novel effective therapeutic option for patients with RAIR-DTC. TERT and BRAF^V600E^ mutations may represent as biomarker for predicting salutary effects of anlotinib.

## Introduction

The incidence of thyroid cancer has increased rapidly in recent decades. Differentiated thyroid carcinoma (DTC), mainly consists of papillary thyroid carcinoma (PTC) and follicular thyroid carcinoma (FTC), make up more than 90% of all of thyroid cancers (1). Irrespective of excellent prognosis in most DTC patients after standard treatment, including surgery, selective radioactive iodine (RAI) therapy, and thyroid stimulating hormone (TSH) suppression therapy. However, one study found that follow-up of 1,038 patients between 1930 and 1985 revealed distant metastases in 4% of patients (2). About one-third of DTC patients with recurrence or metastasis initially or gradually lose the ability of iodine uptake due to de-differentiation, presenting as a RAI-refractory state (3). Patients with RAIR-DTC have a poor prognosis, with an average life expectancy of only 3–5 years and 5- and 10-year survival rates of 19 and 10%, respectively (3).

RAIR-DTC is insensitive to conventional chemotherapy. Many targeted drugs [including donafenib (4), pazopanib (5, 6), cabozantinib (7), vandetanib (8), axitinib (9), sunitinib (10), motesanib (11)] are increasingly being used to treat RAIR-DTC due to many signaling pathways and gene mutations that driving thyroid tumorigenesis have been identified. Although sorafenib and lenvatinib have been approved by the U.S. Food and Drug Administration to be used in RAIR-DTC, not all patients have access to or are able to afford those drugs (12).

Anlotinib is a new, orally administered tyrosine kinase inhibitor (TKI) that targets vascular endothelial growth factor receptor (VEGFR), fibroblast growth factor receptor (FGFR), platelet-derived growth factor receptors (PDGFR), and c-kit. In a Phase I study, anlotinib showed manageable toxicity and broad-spectrum antitumor potential (13). A phase II clinical trial for the treatment of RAIR-DTC with anlotinib (registration number: NCT02586337) was conducted in China. Fortunately, one patient enrolled in our institution achieved satisfactory results, and the experience of this case is reported.

## Case Presentation

### Treatment Prior to Anlotinib

**Figure 1:**
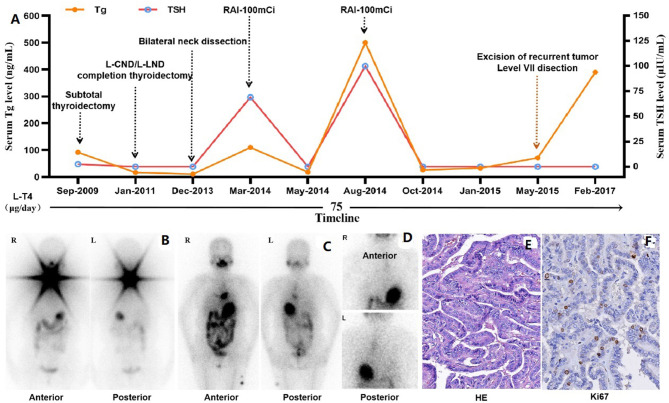
Treatment prior to anlotinib therapy. **(A)** Timeline of treatment prior to clinical study, including four operations in the neck, two sessions of RAI therapy and TSH suppression therapy with L-T4. **(B)** Functional iodine uptake was observed in the neck on the initial post-treatment whole-body scan. **(C)** No functional iodine uptake was observed in the neck on the second post-therapy whole-body scan, but an abnormal radioactive iodine uptake foci in anterior-inferior mediastinum. **(D)** Abnormal radioactive iodine uptake foci in anterior-inferior mediastinum was confirmed to be external contamination by change clothes. **(E)** Papillary thyroid carcinoma was confirmed pathologically with specimen from the 4th operation (HE stain, x200) after RAIR-DTC was developed, and Ki67 stain revealed about 10% positive rate. Tg, Thyroglobulin; TSH, Thyroid Stimulating Hormone; L-CND, Left-Central Neck Dissection; L-LND, Left-Lateral Neck Dissection; RAI, Radioactive Iodine.

A 49-year-old woman was initially diagnosed with PTC in September 2009 in our hospital, and four neck operations and two sessions of radioiodine therapy (RAI) were performed on her till May 2015, and she was diagnosed with RAIR-DTC in August 2014, 7 months prior to last surgery. However, in February 2017, she was readmitted to our hospital for surgical treatment of recurrent tumor in the neck. An enhanced CT scan revealed multiple recurrent lesions in the neck, extensive pulmonary metastases, and metastases behind the left scapula, which could not be completely resected. She refused sorafenib therapy due to the high cost of treatment, and was willing to participate in a phase II, double-blind, placebo-controlled, multicenter clinical trial of anlotinib in RAIR-DTC (registration number: NCT02586337). Treatment prior to anlotinib clinical trial is summarized in the following figure (Figure 1).

### Anlotinib Therapy

A double-blind, randomized drugs (Jiangsu Chia-tai Tianqing Pharmaceutical Co. Ltd.) for anlotinib clinical trial was administrated in March 2017, 12 mg p.o. daily with a 2-week on/1-week off regimen. The drug protocol was chosen from previous study (13). Efficacy was evaluated every two cycles by CT scan, progression was observed at C8 and revealed that the patient was taking placebo. She was referred to the open label therapy of anlotinib in September 2017, 12 mg p.o. daily with a 2- week on/1-week off regimen. Imaging evaluation was performed before C12 in every two cycles and after C12 in every four cycles.

### Efficacy

Based on baseline CT imaging evaluation, the tumors on the right anterior sternocleidomastoid muscle, under the right subclavian and left posterior scapula were chosen as the target lesions, while the other recurrent tumors in the neck and extensive lung metastases were as non-target lesions.

**Figure 2:**
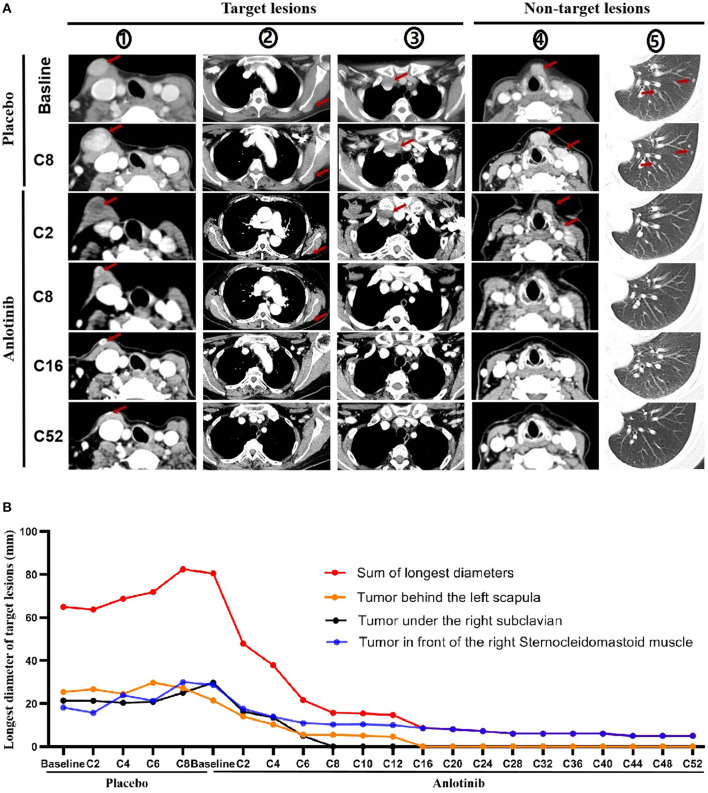
Tumor shrinkage during placebo and anlotinib therapy. **(A)** Imaging evaluation on target and non-target lesions, including placebo therapy (baseline, C2, C8) and anlotinib therapy (baseline, C2, C8, C16, and C52). (①: Tumor under the right subclavian; ②: Tumor in front of the right sternocleidomastoid muscle; ③: Tumor behind the left scapula; ④: Tumor in front of larynx and tumor in the cervical sheath; ⑤: Metastatic tumor of the left lung). Red Arrow: tumor. **(B)** Tumor shrinkage per review (Evaluation was performed before C12 in every two cycles and after C12 in every four cycles).

Progression occurred at C8 with placebo treatment (Figure 2A). Partial response was achieved at C2 of open label treatment with anlotinib, the sum of the largest diameter of target lesions reduced by 38.26%, the non-target lesions in the neck also shrank significantly, and the pulmonary micro-metastases disappeared. Target tumor under the right subclavian and other non-target lesions disappeared at C8 and tumor in front of the left posterior scapula also disappeared at C16. So far, this patient has been treated with 52 cycles of anlotinib at the time of manuscript preparation, with a reduction of 93.79% in the target lesions, and no new lesions emerged (Figure 2A). Tumor shrinkage during placebo and anlotinib therapy are shown in figure below (Figure 2B).

### TSH Suppression Therapy

**Figure 3:**
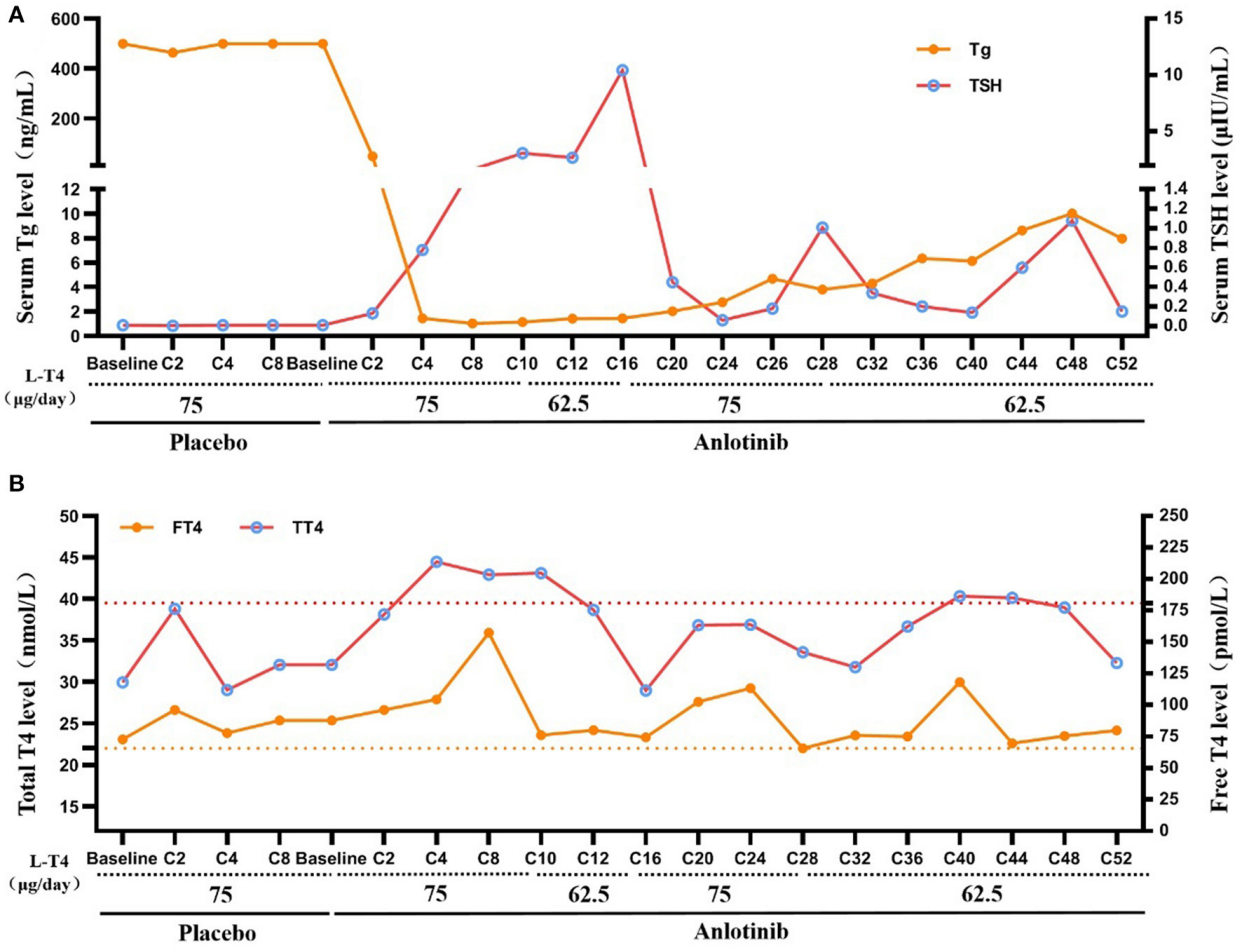
TSH suppression therapy during anlotinib treatment. **(A)** Change of serum TT4 and FT4 during placebo and anlotinib therapy (Yellow dotted line: upper limit of FT4, Normal range: 12–22 pmol/L; Red dotted line: upper limit of TT4, Normal range: 66–181 nmol/L). **(B)** Change of serum TSH and Tg during placebo and anlotinib therapy (TSH normal range: 0.27–4.2 μIU/mL; Tg normal range: 3.5–77 ng/mL).

TSH suppression therapy with L-T4 was continued during anlotinib therapy (Figure 3). TSH was maintained between 0.05 and 0.1 μIU/mL with 75 μg/d of L-T4 prior to clinical trial and during placebo treatment. However, during anlotinib treatment, the dose of L-T4 was adjusted between 62.5 and 75 μg daily, which was maintained at 62.5 μg since C28 with slightly elevated FT4.

Serum Tg level was consistently higher than 500 ng/mL during placebo treatment. After treatment with anlotinib, the serum Tg level decreased sharply to 48.24 ng/mL at C2, and then to the lowest level (1.03 ng/ml) at C8. However, it increased gradually from then on to 7.96 ng/ml at C52. The patient has consistently negative serum TgAb since 2009.

### Adverse Effect

During placebo treatment, this patient experienced adverse events (AE) such as hypertension, tachycardia, and twitching of the hand and foot, but all were grade 1. The AEs during anlotinib treatment increased, but most of them could be controlled by drugs, and a few disappeared without treatment. AEs of grade 3 include diarrhea, hypertension, hand-foot syndrome, and headache. Hypertension and hand-foot syndrome persisted throughout the study. No grade 4 or grade 5 AE was recorded. Eastern Cooperative Oncology Group (ECOG) performance status was 1 at every review during anlotinib therapy, which was 0 on baseline and placebo therapy.

### Molecular Analysis

This patient did not take a molecular diagnosis until favorable outcome with anlotinib therapy. Tumor-specific genetic changes using next-generation sequencing (NGS) panel covers 50 genes was performed for the 4th surgical tissue. The results showed the TERT p.C228T mutation [minor allele frequency (MAF) = 36.14%, formalin-fixed, paraffin- embedded sample] and c.T1799A: p.V600E mutation of BRAF gene [minor allele frequency (MAF) = 36.68%, formalin-fixed, paraffin-embedded sample].

## Discussion

Although the majority of patients with DTC have good prognosis, only a few patients have a poor prognosis, especially those with iodine refractory, with a 10 years survival rate of <10% due to no effective treatment (1). The case we reported who underwent four operations and two sessions of RAI treatments, and was diagnosed with RAIR-DTC. Despite long-term TSH suppression therapy (TSH <0.01 μIU/mL), the tumor still relapsed in the neck and subsequent metastasized to lungs and others distant site. The poor prognosis of this patient can be predicted, and effective treatment is imminent. This patient has no access to lenvatinib or cannot afford sorafenib therapy, which were approved to be used in RAIR-DTC by US FDA and can improve progression-free survival (PFS) of RAIR -DTC by 14.7 months (14) and 5 months (15), respectively. Eight cycles of placebo therapy revealed the PFS of this case was about 6 months, indicating the highly invasiveness nature of the tumor. Luckily, when she converted to anlotinib open-label therapy, excellent response was achieved.

Anlotinib is a multitarget tyrosine kinase inhibitor that was originally designed to inhibit VEGFR2/3, FGFR1-4, PDGFRα/β, c-Kit, and Ret (16), thereby exerting inhibitory effects on tumor angiogenesis and growth (13), tumor invasion (17), lymphangiogenesis and lymphatic metastasis (18). Anlotinib has been shown to be effective in the treatment of advanced non-small cell lung cancer (19), small cell lung cancer (20), esophageal squamous cell carcinoma (21), metastatic colorectal carcinoma (22), soft tissue sarcoma (23). Anlotinib has also demonstrated a sustained antitumor activity in locally advanced or metastatic medullary thyroid carcinoma, with a PFS rate of 85.5% at 48 weeks of treatment (24). Preclinical studies have shown that anlotinib inhibits the cell viability of papillary thyroid cancer and anaplastic thyroid cancer cell lines, and suppresses the migration of thyroid cancer cells *in vitro* and the growth of xenograft thyroid tumors in mice (25). Anlotinib has significant anticancer activity in thyroid cancer, and potentially offers an effective therapeutic strategy for patients of advanced thyroid cancer type.

In the case we reported, the disease progression occurred at C8 of placebo treatment, indicating the highly invasiveness nature of the tumor, which is consistent with dual mutation in TERT and BRAF^V600E^ genes. Inconsistent PFS (10.8, 11.1, 12.7, 15.1, 14.98, 18.3 months) (4, 7, 14, 15, 26)were reported in clinical trials of locally advanced or metastatic RAIR-DTC treated with multikinase inhibitors (MKIs) or TKIs, partially due to different entry criteria, and when partial response will be achieved were unknown (4, 7, 14, 15, 26). Atractingly, partial response was achieved at C2 of anlotinib treatment at 12 mg p.o. daily with a 2-week on/1-week off regimen, and not only the target tumor in the neck, but also the distant target lesion and the non-target lesions in the lung showed excellent response. Extensive pulmonary micro-metastases disappeared at C2, and serum Tg decreased sharply from more than 500 ng/mL to 48.24 ng/mL, which also showed an immediately satisfactory biochemical remission. Such encouraging result suggests that two cycles of treatment could be a litmus test for the effectiveness of anlotinib against RAIR-DTC. As the treatment progressed, the target lesion shrank continuously, and the non-target lesion disappeared at C16, serum Tg levels dropped to the lowest level (1.03 ng/ml) at C8, and target lesions shrank by 93.7% at C52, suggesting that long-term anlotinib therapy has not developed resistance.

Since TSH suppression therapy does not conflict with anlotinib therapy, TSH suppression therapy was continued. TSH was slightly elevated in the early stages of anlotinib treatment with 75 μg/day of L-T4. However, the dose of L-T4 could be reduced to 62.5 μg/d since C28, indicating the distribution or utilization of L-T4 might be influenced by anlotinib, it also reminds clinicians to be aware of this problem and implement measures to minimize the occurrence of overdosing and the potential for long-term complications. Interestingly, Bible et al. (5) observed TSH levels increased by >15% between baseline and cycle one/4 weeks reassessment in 64.6% of RAIR-DTC patients treated with pazopanib, indicating that early TSH increment is frequent in response to pazopanib therapy.

Finally, the safety of treatment is as important as its effectiveness. Although sorafenib could improve the PFS of the RAIR-DTC, a recent systematic review by Feng et al. concluded that the use of sorafenib should be cautious due to a high incidence of adverse effects (27). Gao et al. (28) systematically reviewed the clinical studies on the safety valuation and management of adverse events associated with the clinical use of anlotinib for cancer treatment, and concluded that the toxicities of anlotinib were acceptable or manageable in clinical trials and real-world clinical cases of patients with advanced cancers. No SAEs occurred in this patient, most of which could be controlled with medication, and a few of which could disappear spontaneously, with grade 3 AEs including diarrhea, hypertension, and headache. Interestingly, hypertension was considered to be a biomarker for efficacy prediction of anlotinib in advanced NSCLC (19). Hand-foot syndrome persists and affects the patient's quality of life, and also can be relieved by taking celecoxib and applying urea-vitamin E cream. Anlotinib demonstrated a manageable adverse event profile in this patient with RAIR-DTC. The relationship between side effects and efficacy is worth further study.

Previous research has shown that various molecular pathways play important roles in tumorigenesis and development, including proliferation pathway, cell cycle control pathway, and angiogenesis process (29). Anlotinib plays important roles in intracellular tyrosine phosphorylation and intracellular signaling (30). In our case report, the patient carried TERT promoter and BRAF^V600E^ mutations according to the NGS analysis. It is well-known that both TERT promoter and BRAF^V600E^ mutations are associated with develop and poor prognosis of RAIR-DTC. Several studies further revealed that coexistent BRAF^V600E^ and TERT promoter mutations have a synergistic effect on poor clinical outcomes (31). Our case is the first report in the world in which a patient with recurrent RAIR-DTC harboring TERT p.C228T mutation and BRAF^V600E^ mutation was treated with anlotinib, and the patient achieved a partial response that has been maintained for 37 months. Based on these preliminary data, we speculated that coexistent TERT p.C228T mutation and BRAF^V600E^ mutation might be a biomarker to predict the beneficial effect of anlotinib. This also reflects the importance of molecular diagnosis for clinically complex cases.

Limitations are that we cannot explain the significance of the imaging evaluation of the progressive increase in serum Tg levels after C8 and the persistent shrinkage of tumors in target and non-target areas, mainly because this clinical trial of ours conflicts with diagnostic whole-body iodine imaging or PET-CT scans, which cannot detect whether a patient has recurred and can only be determined by examination after the patient has withdrawn from the trial; and for the effect of anlotinib on L-T4 metabolism *in vivo* mechanism, we also only consider that anlotinib affects the protein function within the thyroid tissue, which leads to the uneven distribution of TT4 and FT4 *in vivo*. Therefore, it is necessary to collect more cases and perform further studies to clarify these issues.

## Conclusion

In conclusion, we have reported the first case of RAIR-DTC patients with co-existing TERT p.C228T mutation and BRAFV600E mutation treated with anlotinib. In this report, RAIR-DTC progresses rapidly, anlotinib demonstrated promising efficacy (PR can be achieve at C2 and long-term treatment did not show resistance) as well as manageable adverse event profile in this patient, which suggests that anlotinib might provide a novel effective therapeutic option for patients with RAIR-DTC. TERT p.C228T mutation and BRAF^V600E^ mutation might be the underlying target of anlotinib. Currently, drug selection is applied primarily on the basis of patient characteristics and expected toxicity indicators, with only one drug having a potential predictive effect. In the future, the challenge is to design feasible drugs for DTC patients with concurrent genetic mutations based on the target of the corresponding mutation and to minimize toxicity, leading to precision therapy with maximum patient benefit.

## Data Availability Statement

The raw data supporting the conclusions of this article will be made available by the authors, without undue reservation.

## Ethics Statement

The patient has signed her written informed consent for clinical study and publishing the case details.

## Author Contributions

RC had full access to the data and took final responsibility for the decision to submit for publication. All authors contributed to the article and approved the submitted version.

## Conflict of Interest

The authors declare that the research was conducted in the absence of any commercial or financial relationships that could be construed as a potential conflict of interest.
